# Computed Tomography-Based Investigation on the Effects of Intravenous Bisphosphonate Administration on Tooth Growth in a Minipig Animal Model

**DOI:** 10.3390/medicina58060778

**Published:** 2022-06-09

**Authors:** Philipp Poxleitner, Pit J. Voss, David Steybe, Lisa-Marie Seibert, Stephan Zeiter, Martin J. Stoddart, Rainer Schmelzeisen, Sven Otto

**Affiliations:** 1 Department of Oral and Maxillofacial Surgery, Medical Center—University of Freiburg, Faculty of Medicine, University of Freiburg, 79106 Freiburg, Germany; pit.voss@uniklinik-freiburg.de (P.J.V.); david.steybe@uniklinik-freiburg.de (D.S.); lisa_marie_seibert@yahoo.com (L.-M.S.); rainer.schmelzeisen@uniklinik-freiburg.de (R.S.); 2 AO Research Institute Davos, Clavadelerstrasse 8, 7270 Davos, Switzerland; stephan.zeiter@aofoundation.org (S.Z.); martin.stoddart@aofoundation.org (M.J.S.); 3 Department of Oral and Maxillofacial Surgery, Ludwig-Maximilians-University Munich, Lindwurmstr. 2a, 80337 Munich, Germany; sven.otto@med.uni-muenchen.de

**Keywords:** bisphosphonates, minipig, tooth development, tooth growth

## Abstract

*Background and Objectives*: The objective of this study was to evaluate the effects of bisphosphonate (BP) administration on tooth growth, using CT-data of a minipig animal model investigation. *Materials and Methods*: Tooth growth was evaluated in minipigs, with eight animals receiving weekly zoledronate (ZOL) and three animals serving as the control group. Tooth growth was evaluated at the right 2nd molar (M2) in the maxilla. A computed tomography-based measuring method was applied to evaluate tooth growth in the coronal-apical, buccal-oral and mesial-distal axis. *Results*: ZOL-administration was found to impact tooth growth in all evaluated measuring axes, with the highest effect observed in the coronal-apical axis. *Conclusions*: Detrimental effects of BP administration on growing teeth have been reported by a number of investigators. The results of this investigation demonstrate that intravenous ZOL affects the growth of the whole tooth within a short period of administration. With BPs being administered to a growing number of pediatric patients, further studies should be conducted to qualify and quantify the effects of BPs on developing teeth.

## 1. Introduction

Bisphosphonates (BPs) are a major class of antiresorptive agents and frequently administered in cases of bone diseases associated with excessive bone resorption/bone turnover. They bind covalently to hydroxyapatite crystals of the bone and are incorporated into the osteoclast during bone resorption [1]. BPs can be categorized into non nitrogen-containing BPs (non-N-BPs) and nitrogen-containing BPs (N-BPs), with zoledronate being a representative of the latter group. N-BPs act by targeting farnesyl pyrophosphate synthase in the mevalonate pathway. The inhibition of this enzyme prevents the modification of important signaling proteins, evoking the inhibition of osteoclast differentiation and function and increased apoptosis [2,3]. These processes ultimately decrease bone resorption and remodeling.

Being an established part of the medical treatment of conditions associated with excessive bone resorption in adult patients for many years, more recently, pediatric indications for BP treatment have emerged. BPs are now increasingly used in children and adolescents for the treatment of skeletal pediatric disorders such as osteogenesis imperfecta, juvenile idiopathic osteoporosis and other diseases associated with excessive bone resorption [4]. Due to the relatively recent use of BPs in children, compared with adults, there is a paucity of data on the potential side effects of BP administration on growing bone and teeth. The main effect of BP administration is the inhibition of osteoclasts with a subsequent decrease in bone turnover [5]. The eruption of teeth requires the resorption of the alveolar bone coronal to the developing tooth and, with the exception of permanent molars, resorption of primary teeth. Administering BPs might thus interfere with tooth exfoliation and the eruption process, an assumption that has been confirmed by a number of animal and clinical studies reporting delayed or inhibited tooth eruption under BP treatment [6,7,8,9]. Moreover, the administration of BPs might have a direct effect on dental tissues; however, to date, this aspect has been investigated by a low number of in vitro and animal studies only [7,8,10,11].

In this context, the aim of the present investigation was to assess the impact of intra-venous ZOL administration on tooth-growth in a minipig animal model. Unlike previous studies on the effects of BP administration on growing teeth, we used a computed tomography (CT)-based approach with CT-scans performed at different times during the course of BP administration in order to evaluate the effects of intravenous BP administration on growing teeth in different measuring axes. A minipig animal model was chosen as the porcine jawbone, teeth and oral microflora are reported to closely resemble those of humans [12,13].

## 2. Materials and Methods

All procedures of this study were performed at an AAALAC accredited facility according to the Swiss Laws of animal welfare and were approved by the local Animal Welfare Commission of the official veterinary authorities (Office for food safety and animal health Graubünden, Approval Code: TVB 23/2013). This present investigation is based on the CT-data of a BRONJ animal model study conducted on minipigs at the AO research institute, Davos, Switzerland. A veterinary examination confirmed all animals to be healthy at the time of initiating the study. All animals underwent an acclimatization period of 4 weeks before initiating the study procedures with the administration of weekly ZOL infusion. During this period, the minipigs were trained for the study procedures, e.g., blood withdrawal and weight measurements. Animals were kept in group housing on deep straw with access to the outside and fed with pellets (Maintenance feed, Provimi Kliba AG, Kaiseraugst, Switzerland). ZOL administration (0.05 mg/kg bodyweight once per week) and sedation were performed as previously published [14].

**Figure 1:**
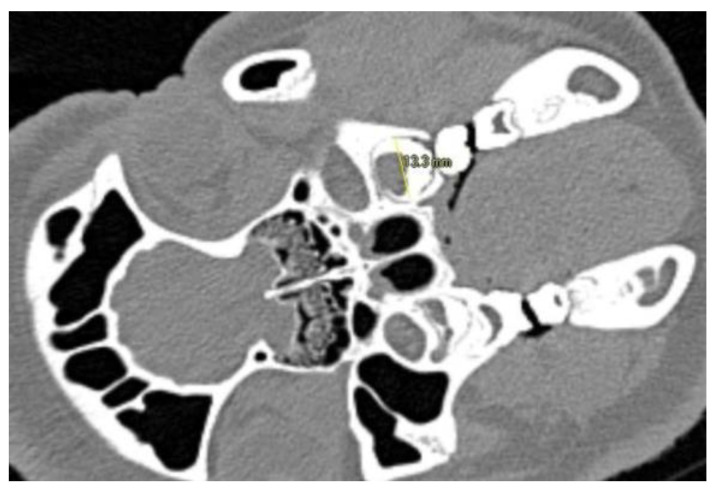
Visualization of the measuring procedure in CT-slices. Figure depicts measurement in buccal-oral axis. The outer contour of the tooth is determined as the interface between the hyperdense structure of the tooth and the hypodense structure of the desmodontal gap.

The aim of this preliminary investigation was to assess the impact of ZOL administration on tooth growth, based on CT-data of the BRONJ animal model investigation and due to this study design, no formal sample size calculation was performed. In the context of this investigation, the animals were divided into two groups. Group 1 (n = 3) comprises the animals that did not receive ZOL and thus serves as a control group. Group 2 (n = 8) includes all animals that were administered ZOL as part of the BRONJ animal investigation. Tooth growth of the 2nd molar (M2) was evaluated using computed tomography (CT) scans performed at two different times during the BP administration period (CT_1_ and CT_2_). CT-data were acquired using a Siemens SOMATOM Emotion 6 (Siemens, Erlangen, Germany) with a tube voltage of 130 kVp and tube current of 125 mA. The acquired images had a slice thickness of 0.63 mm and a resolution of 0.5 mm. CT_1_ was performed at the time of initiating weekly BP administration in all animals. CT_2_ was performed after a mean duration of a further 17 weeks of ZOL administration (range 12–19 weeks). The size of the second maxillary molar at the time of CT_1_ and CT_2_ was evaluated using VoXim Osteo 6.0 (IVS Solutions AG, Germany) by determining the outer contour of the tooth as the interface between the hyperdense structure of the tooth and the hypodense structure of the desmodontal gap (Figure 1). Each tooth was measured in three sectional planes, with measurements performed in two axes per plane (Tabel 1); for better comparability, we delimited the measuring ranges using a graphic plateau selection.

### Statistical Anlysis

A statistical analysis revealed an intraclass correlation coefficient (ICC) of 0.9944 for observer 1, an ICC of 0.9946 for observer 2 and an ICC of 0.9936 when comparing the results obtained by the two observers. The measurements were summarized as a mean value, the mean values of the corresponding measuring axes in the three sectional planes (see Table 1) were summed up. Tooth growth was then determined by calculating the difference between the tooth size at the time of CT2 and the tooth size at the time of CT1. This value was divided by the observation time (time between CT_1_ and CT_2_), resulting in the tooth growth given in mm/day. These results were collected in a Microsoft Excel spreadsheet and statistically processed by transferring them into the statistical data analysis software JMP PRO Version 13 (SAS Institute Inc., Cary, North Carolina, USA). The level of significance was set at 5% (*p* ≤ 0.05) in this statistical analysis. Significant values are marked with an asterisk in the following graphs. The group mean values were statistically compared by applying an analysis of variance (ANOVA).

## 3. Results

**Figure 2:**
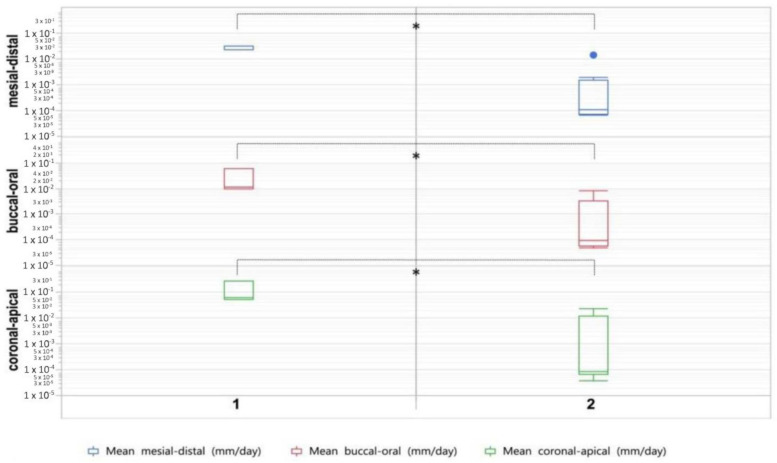
Box plots showing tooth growth in minipigs not administered ZOL (**1**) and minipigs administered ZOL (**2**). Asterisk indicates statistically significant differences (*p* ≤ 0.05).

In this study, we investigated the effects of BP administration on growing teeth, based on the CT-data of a BRONJ animal model investigation. Eleven minipigs were included into the study; and the mean age of the animals at the time of CT_1_ was 333 days and 428 days at the time of CT_2_. Evaluating the change in tooth size between CT_1_ and CT_2_, we found considerably lower growth values in the animals administered ZOL. It is worth noting that the observed changes occurred within a mean duration of just 17 weeks. The results are depicted in Table 2 and Figure 2. In the coronal-apical axis, the group values differ significantly (*p* = 0.013). A mean tooth growth of 0.123 mm/day (±0.117) was found for group 1. In group 2, we observed a mean tooth growth of 0.005 mm/day (±0.009). Significantly different group values (*p* = 0.022) were also found in the buccal-oral axis. A mean tooth growth of 0.027 mm/day (±0.028) is found in group 1. In group 2, we observed a mean tooth growth of 0.002 mm/day (±0.003). Significantly different group values (*p* = 0.0001) were found in the mesial-distal axis too. A mean tooth growth of 0.029 mm/day (±0.006) was found in group 1. In group 2, we observed a mean tooth growth of 0.002 mm/day (±0.005).

## 4. Discussion

With BPs introduced into pediatric medicine more recently and these agents being administered for a growing number of pediatric conditions associated with excessive bone resorption, the potential side effects of this medication on growing bone and dental tissues are of particular interest. The side effects on odontogenesis might originate from the indirect effects of suppressed bone turnover on developing teeth as well as from direct effects of BPs on teeth-forming cells and dental tissues. In this present investigation, we were aiming at evaluating the effects of ZOL administration on tooth growth in a minipig animal model and based on CT-data. A minipig animal model was chosen as the porcine jawbone structure and metabolism, teeth and oral microflora are reported to closely resemble those of humans [12,13,15,16,17]. The advantage of the applied method is the differentiated statement on tooth-growth in different oriental directions in three-dimensional space. In the literature, tooth size is often described by the mesio-distal tooth width [18]. This corresponds to the mesio-distal measurement axis in this study.

Delayed root development (i.e., changes in coronal-apical tooth growth) was reported by Bradaschia-Correa et al. [7], Hiraga et al. [8] and Lézot et al. [19]. Unlike these investigators, we used CT-scans performed at different times during the BP administration period to evaluate tooth growth in different oriental directions. This way, we could demonstrate that BPs not only affect tooth-growth in the coronal-apical axis but in the buccal-oral and mesial-distal axis as well. Moreover, this effect seems to occur within a short period of BP administration with the changes in tooth growth observed in this study within just 17 weeks. The effects of BP administration on odontogenesis have been investigated in a number of in vitro, animal and clinical studies. Bradaschia-Correa et al. demonstrated inhibitory effects of BP medication on tooth eruption in growing rats [7]. Correspondingly, Grier and Wise [20] as well as Hiraga et al. [8] reported a delayed eruption of incisors and molars in BP-treated rats. Only limited clinical data are available on this aspect; however, these investigations support the results reported in animal studies [4,6,9]. Besides the inhibitory effects of BP medication on tooth eruption, Bradaschia-Correa et al. observed the occurrence of ankylosis, injuries to the dental epithelium and lack of root formation [7]. A relation between BP administration and the induction of several types of dental abnormalities in rats, including defects of the enamel, was observed with ZOL administration as well [8]. Although these authors concluded that the ZOL induced dental abnormalities observed in their study may be explained entirely by the BP’s impact on osteoclastic bone resorption, they suggest direct effects of ZOL on odontogenic cells to be investigated [8]. Investigations on this aspect are sparse; however, there are data available on the dose-dependent cytotoxic effects of ZOL on cultured odontoblast-like cells (MDCP-23) [10].

It can be concluded from the current literature that BPs have detrimental effects on odontogenesis. These findings may be attributed to the indirect effects of reduced bone turnover/resorption as well as the direct effects of BPs on odontogenic cells. Moreover, molecular signaling between bone cells and dental/periodontal cells, as proposed by Gama et al. might play a role [21]. The small sample size, particularly the small control group, can be considered a limitation of this study. However, the results obtained from the control group demonstrated a growth pattern that would be expected under physiologic conditions, with the highest growth-rate observed in the coronal-apical axis. The highest impact of ZOL administration was found in this axis and this observation is in accordance with previous studies that reported inhibitory effects of BP administration on root development/tooth eruption. However, the application of a CT-based approach allowed us to evaluate tooth growth in the buccal-oral and mesial-distal axis as well. Although lower than in the coronal-apical axis, BP administration had considerable, statistically significant effects on tooth growth in these axes as well. These findings suggest BP administration impacts not only tooth eruption but the development of the tooth as a whole. Further investigations are necessary to quantify and qualify the exact influence of BPs on tooth growth.

## 5. Conclusions

BPs are administered for a growing number of conditions associated with excessive bone resorption in pediatric patients and detrimental effects of BP administration on growing teeth have been reported by a number of investigators. Although no definitive conclusion can be drawn from the results of this preliminary study, the findings of this investigation suggest BPs to influence the development/growth of the tooth as a whole and indicate that these changes occur within a short period of BP administration. As BPs are administered for a growing number of conditions associated with excessive bone resorption in pediatric patients, further investigations on a larger sample should be performed to quantify and qualify the effects of BPs on developing teeth.

## Figures and Tables

**Table 1:** Overview of sectional planes and measurement axes.

Sectional Plane	Measurement Axis	Anatomical Orientation
Axial	coronal-apical	Parallel with nasal septum and central through tooth
	buccal-oral	Parallel with palatal vault and at the level of the alveolar margin
Coronal	mesial-distal	Central at the level of the alveolar ridge and central through the tooth
	buccal-oral	Orthogonal with palatine suture and central through tooth
Sagittal	mesial-distal	Central through pulp cavity
	coronal-apical	Orthogonal with alveolar ridge and central through tooth

**Table 2:** Mean tooth growth in mm/day for the three measuring axes evaluated in this investigation.

	Group 1 (No ZOL-Administration)	Group 2 (ZOL Administration)	*p*-Value
Mesial-distal	0.029 ± 0.006	0.002 ± 0.005	0.0001
Buccal-oral	0.027 ± 0.028	0.002 ± 0.003	0.022
Coronal-apical	0.123 ± 0.117	0.005 ± 0.009	0.013

ZOL = zoledronate.

## Data Availability

The data presented in this study are available on request from the corresponding author.
